# Recumbent FES‐Cycling Exercise Improves Muscle Performance and Ambulation Capacity in Hospitalized Patients: A Randomized Controlled Trial

**DOI:** 10.1111/aor.15029

**Published:** 2025-05-31

**Authors:** Murillo Frazão, Fábio de Lima Martins, Gerson Cipriano

**Affiliations:** ^1^ Lauro Wanderley University Hospital – UFPB/EBSERH João Pessoa PB Brazil; ^2^ Postgraduate Program in Health Sciences and Technologies University of Brasília – UnB Brasília Brazil

**Keywords:** ambulation, efficiency, electrical stimulation, exercise, FES‐cycling, power, strength, torque, weakness

## Abstract

**Background:**

Acquired muscle weakness is a prevalent complication during hospitalization. Supportive technologies, such as functional electrical stimulation cycling (FES‐cycling), are increasingly recognized as a tool with the potential to improve physical exercise in patients constrained to bed rest.

**Methods:**

In this randomized clinical trial, patients admitted to a high‐complexity ward exhibiting clinical signs of muscle weakness (e.g., report of loss of strength, gait, or balance deficit due to weakness or restriction to bed) were enrolled. Participants were randomly allocated to a recumbent high‐intensity, low‐volume FES‐cycling exercise or a control group. The primary outcomes measured were torque, power output, stimulation cost (neuromuscular efficiency), and ambulation capacity.

**Results:**

The analysis included 16 patients (eight in each group). Postintervention, the FES‐cycling group presented a greater increase in both absolute (4.25 ± 3.15 vs. 0.04 ± 3.49 Nm, *p* = 0.02) and percentage torque (117 ± 88 vs. 8% ± 53%, *p* < 0.01) compared to the control. Similarly, the FES‐cycling group presented higher absolute (3.91 ± 2.25 vs. 0.57 ± 1.82 watts, *p* < 0.01) and percentage power (61 ± 36 vs. 10% ± 23%, *p* < 0.01), along with a higher absolute (−2903 ± 2598 vs. −523 ± 1319 μC/watt, *p* = 0.03) and percentage stimulation cost (−33 ± 18 vs. −6% ± 1 8%, *p* = 0.01). Additionally, enhanced ambulation capacity was observed in the FES‐cycling group, with 6 patients showing improvement versus 2 in the control group (*p* = 0.03).

**Conclusions:**

Recumbent high‐intensity, low‐volume FES‐cycling exercise increased muscle strength, power, and neuromuscular efficiency in hospitalized patients with muscle weakness. Improvements in ambulation capacity were also noted, supporting the intervention potential.

## Graphical abstract

Both groups received the usual routine hospital care. Beyond usual care, the intervention group received the high‐intensity, low‐volume FES‐cycling exercise protocol. Study highlights are as follows: (1) High‐intensity, low‐volume FES‐cycling exercise increased strength, power, and neuromuscular efficiency. (2) Ambulation capacity improved threefold in the FES‐cycling exercise group compared to the control.

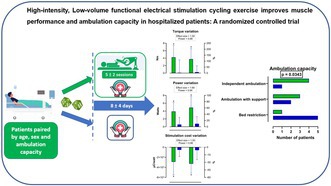

## 1 Introduction

Muscle weakness is a common comorbidity during hospitalization, especially in the intensive care unit (ICU). Frequently, weakness arises as a secondary condition during treatment for other critical illnesses, referred to as ICU‐acquired weakness. This condition is typically generalized, symmetrical, and affects proximal limb and respiratory muscles, sparing facial and ocular muscles. Muscle tone is often reduced, and reflexes may be normal or diminished. ICU‐acquired weakness can result from critical illness polyneuropathy, critical illness myopathy, or both (critical illness neuromyopathy). Electrophysiological tests show characteristic abnormalities, and significant muscle mass loss, which can exceed 10% in the first week, is linked to functional impairment. Severe disuse muscle atrophy, without electrophysiological abnormalities, is considered a separate form of ICU‐acquired weakness [1].

This dysfunction is often linked to the inflammatory responses but can occur independently of the underlying pathology leading to hospital admission. Prolonged bed rest significantly impacts neuromuscular control and muscle protein synthesis [2]; in fact, just 5 days of bed rest can lead to a marked decrease in neuromuscular efficiency, reducing the strength generated for the same level of neural activation [3]. Additionally, short‐term bed rest can also reduce the cross‐sectional area across all fiber types, decrease capillary density, and disrupt the maintenance of muscle mass and physical function due to increased insulin resistance [2].

The pathophysiological changes associated with bed rest can severely reduce muscle performance and impair ambulatory capacity. On the other hand, early rehabilitation is associated with a decreased likelihood of developing acquired weakness [4]. Physical exercise and neuromuscular electrical stimulation (NMES) interventions have proven effective in preventing excessive muscle mass loss and enhancing muscle strength in patients with acquired weakness [5]. Notably, combining exercise and NMES intervention through supportive technologies such as Functional Electrical Stimulation cycling (FES‐cycling) can overcome barriers to performing exercises, mainly in scenarios restricted by bed confinement [6]. FES‐cycling, which combines computer‐driven electrical pulses delivered by transcutaneous electrodes with a cycle ergometer, promotes muscle contractions and cycling exercise independent of the functionality of the physiological pathway [7].

Studies showing the most significant results in muscle performance outcomes have employed high‐intensity FES‐cycling exercise protocols [8, 9, 10]. However, these studies were not conducted in hospitalized patient populations. Conversely, previous work did not consider high‐intensity level exercise in hospitalized patients [11, 12]. Nevertheless, the safety of recumbent high‐intensity (highest tolerable electrical charge) FES‐cycling exercise protocols in hospitalized patients has recently been demonstrated, showing no muscle damage or clinical instability, especially when combined with a low‐volume (short duration exercise) protocol [13]. One of the key rationales for using a high‐intensity, low‐volume tolerance protocol is the generally low tolerance to exercise programs in this population.

According to the overload principle of training, exercise performed below a certain intensity threshold will not sufficiently challenge the body to induce improvements in physiological parameters [14]. High‐intensity exercise refers to physical activity conducted at a significantly high level of effort, typically near or at the individual's maximum capacity. It is characterized by shorter durations but greater intensity, often resulting in rapid increases in heart rate and energy expenditure. Low‐volume exercise generally refers to workout routines with a shorter overall duration compared to standard or high‐volume exercise programs. It typically involves training sessions of ≤ 10 min of intense exercise [15]. This approach is designed to minimize time commitment while still promoting fitness and strength gains.

Therefore, the primary aim of this study is to establish the effects of a recumbent high‐intensity, low‐volume FES‐cycling exercise on muscle performance (strength, power, and neuromuscular efficiency) in hospitalized patients. The secondary aim is to determine the effects of this exercise regimen on ambulation capacity.

## 2 Methods

This study was conducted in strict accordance with the Consolidated Standards of Reporting Trials (CONSORT) [16]. Ethical approval was granted by the local ethics committee (CAAE 68227323.4.0000.5183, opinion number 6.055.353), and the study was registered on the Brazilian Clinical Trial Registration Platform (RBR‐7bcnt96).

### 2.1 Study Design

This is a randomized controlled trial. Initially, patients who met the inclusion criteria were matched based on age, sex, and ambulation capacity to ensure sample homogeneity. Following this, they were randomly assigned to either the FES‐cycling group or the control group using opaque sealed envelopes in a 1:1 ratio. A single evaluator was responsible for sample matching, randomization, and allocation. Each participant underwent an initial evaluation, received their assigned intervention, and was reevaluated postintervention with identical elapsed times between assessments in both groups.

### 2.2 Participants

Patients admitted to a high complexity ward were enrolled. The data were collected from November 16th, 2023 to April 28th, 2024. Patients who presented clinical signs of muscle weakness (report of loss of strength, gait or balance deficit due to weakness or restriction to bed), of both sexes and over 18 years of age, were included. Patients who did not have clinical conditions for FES‐cycling exercise (hemodynamic instability, resting tachycardia, oxygen saturation < 90% even under oxygen therapy or orthopedic limitations) were excluded. Patients who received less than 3 FES‐cycling exercise sessions or were discharged from the hospital less than 3 days after the initial evaluation were excluded from the analysis.

### 2.3 Ambulation Capacity

Initially, the evaluator asked the patient to get up from the bed and ambulate at least 10 steps. If the patient could ambulate 10 steps without support, they were categorized as having independent ambulation. If the patient needed support (walker, stick, other person assistance, etc.) to ambulate 10 steps, they were categorized as ambulation with support. If the patient could not get up from the bed or ambulate 10 steps, even with support, they were categorized as bed‐restricted. No bed‐restricted patient had a history of being bedbound before hospitalization. Ambulation capacity classification was based on and adapted from two previous studies [17, 18].

### 2.4 Muscle Performance Assessment

The entire evaluation followed the previously described functional electrical stimulation cycling‐based muscular evaluation method [19]. A single expert physical therapist performed all evaluations. The patients were attached to the FES‐cycling equipment (MOBITRONICS, INBRAMED, Porto Alegre, Brazil). Equipment height, distance, and leg support positions were individually adjusted to prevent knee hyperextension and promote a proper range of motion. Self‐adhesive electrodes were placed bilaterally on the belly of the quadriceps (vastus lateralis and vastus medialis), hamstrings, and tibialis anterior muscles and then plugged into the electrical stimulation device cables.

Eight electrical stimulation channels were used. The FES (biphasic, interval, rectangular shape pulse) was set with the same pulse width and current amplitude in all eight channels. Current amplitude was initially set at 40 mA and pulse width at 500 μs. Before the evaluation, the right vastus lateralis channel was activated for 1 s to detect the quality of muscle contraction. The parameters were increased to the pain limit to promote the highest visible muscular contraction. The pain limit was evaluated by self‐reporting (the patient was instructed to inform us when they could no longer tolerate the electrical charge increase). The same parameters were used at baseline and postintervention evaluations.

The FES was triggered (ON) and stopped [20] by the crank position. The equipment has a sensor to detect the 360° crank position, and the FES trigger/stop was set according to joint positions during the cycling movement. In one leg, quadriceps channels (vastus lateralis and vastus medialis) were triggered at around 90° of hip and knee flexion and stopped at around 10° hip flexion and 160° knee extension. In the opposite leg, the hamstrings and tibialis anterior channels were triggered at around 30° of hip and knee flexion and stopped at around 75°.

The equipment was set in the evaluation mode to perform an automatic preset combination (unchangeable) of different cycle ergometry cadences (rotations per minute—rpm) and electrical stimulation frequencies (1st = 10 rpm and 50 Hz, 2nd = 10 rpm and 75 Hz, 3rd = 10 rpm and 100 Hz, 4th = 15 rpm and 50 Hz, 5th = 15 rpm and 75 Hz, 6th = 15 rpm and 100 Hz, 7th = 20 rpm and 50 Hz, 8th = 20 rpm and 75 Hz, and 9th = 20 rpm and 100 Hz) maintaining the previously selected pulse width and current amplitude. The patients performed seven cycling movements in each combination. The patients did not undertake any voluntary effort (they were strictly instructed not to make any voluntary effort). All the work was performed using the FES‐cycling equipment.

The equipment recorded torque (newton meter—Nm) and power output (watts—W), in addition to the stimulation cost (microcoulomb/watt—μC/W), during the entire cycle ergometry cadences and electrical stimulation frequency combinations. The equipment software reported maximal torque and power output, as well as the minimal stimulation cost reached. Torque information is generated by the servo motor drive. The servo motor has an auto‐tuning which provides a specific electrical charge to maintain the programmed rotation. The torque calculation is based on the variance of the electrical charge applied to keep the rotation. The servo motor drive also provides the angular velocity. The power output values are achieved by mathematical calculation (torque times angular velocity). The stimulation cost is the total electrical charge (current amplitude times pulse width) delivered by the electrical stimulator divided by the power output.

### 2.5 Intervention

Both groups received the usual routine hospital care: medical assistance, medications, nursing assistance, nutritional support, psychological support, occupational therapy, and physical therapy. Beyond usual care, the intervention group received the recumbent high‐intensity, low‐volume FES‐cycling exercise protocol. Two independent physical therapists took turns carrying out the FES‐cycling exercise sessions. One performed the assessments and exercise sessions, while the other performed only the exercise sessions (blind to the evaluation results). Both were blind to the usual routine hospital care.

**FIGURE 1:**
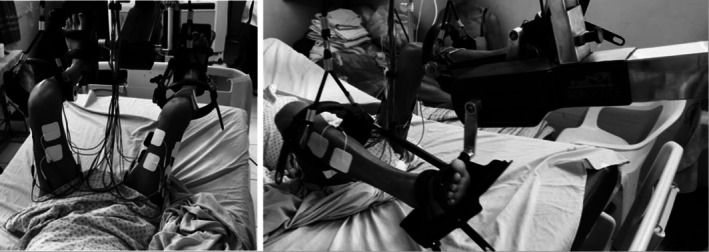
Patient attached to the functional electrical stimulation (FES) cycling equipment.

Patients in the intervention group were attached to the FES‐cycling equipment as described above in the muscle performance assessment (Figure 1). All the FES‐cycling exercise was completed while lying in bed. Self‐adhesive electrodes were placed bilaterally on the belly of the quadriceps (vastus lateralis and vastus medialis), hamstrings, and tibialis anterior muscles. The tibialis anterior was stimulated to prevent/treat hospitalized patients' foot drop [21]. The equipment was placed in the passive FES‐cycling mode (isokinetic, with a pedaling cadence of 15 rpm). Electrical stimulation frequency was fixed at 75 Hz to maximize muscle activation and therapeutic effectiveness. As a high electrical charge is needed in hospitalized patients [22], the highest electrical charge tolerated was applied, respecting the pain limit. Before starting each exercise session, a channel was activated for 1 s to determine the highest pulse width and current amplitude parameters (as described above). Patients underwent 10 min of exercise in each session. Exercise periodization (the planned variation of the physical training variables) was done through current amplitude and pulse width adjustments. Patients were strongly encouraged to allow the physical therapist to gradually increase the electrical charge (current amplitude and/or pulse width) during each session.

The patients received a maximum of 10 FES‐cycling exercise sessions (one session/day) in a maximum 15‐day period. Maximum exercise sessions and intervention days were established to ensure protocol feasibility (based on the hospital routine and length of stay characteristics). Exercise sessions were interrupted before the maximum 10 FES‐cycling exercise sessions or 15 days if the patient was discharged from the hospital, transferred to another hospital sector, or was able to ambulate independently. In this situation, reevaluation was performed on the day of the discharge/transference or independent ambulation. If the patient left the ward before complete reevaluation, intention‐to‐treat analysis was performed for missing data. If the paired patient in the control group was discharged from the hospital or transferred to another hospital sector before the patient in the intervention group, exercise sessions were also interrupted, and reevaluation was performed to ensure the same elapsed time between evaluations in paired patients of both groups.

### 2.6 Outcomes

The primary outcome was the muscle performance improvement. It was assessed by strength (torque—newton meter, Nm), power output (watts, W), and neuromuscular efficiency (stimulation cost—defined as the electrical charge delivery rate per watt of output power [microcoulomb/watt, μC/W]) variations (differences between baseline and postintervention evaluations). Absolute (postintervention − baseline values) and percentage (postintervention values ÷ baseline values − 1 × 100) values were analyzed. Electrical charge was defined as the product of current amplitude times pulse width (μC). The secondary outcome was the ambulation capacity. The number of patients in each category described above was measured. Exercise periodization variables were also analyzed.

### 2.7 Statistical Analysis

Data normality was verified using the Shapiro–Wilk test. According to the data normality distribution, the specific test was performed. The categorical variables were analyzed using the Fisher exact test. Paired *t*‐tests or Wilcoxon tests were used to analyze periodization data differences. Unpaired *t‐*tests or Mann–Whitney tests were used for intergroup differences analysis. The data are presented as means ± standard deviations or medians and interquartile ranges and percentages. The effect size was calculated for the primary outcome by the *T*‐test (difference between two independent means) and post hoc analysis. The percentage variation values were used. The input parameters were total sample size, means and standard deviations, and error probability *α* = 0.05. The effect size points were small (0.2), medium (0.5), and large (0.8) [23]. The chi‐square test for trend was used to compare ambulation capacity. The sample size was calculated (*T*‐test family − difference between two independent means) based on means ± standard deviation values of the stimulation cost variable, with error probability *α* = 0.05 and power = 0.80. The sample size calculation indicated a minimum of 14 patients (seven in each group). A statistical significance value of *p* ≤ 0.05 was set for all analyses. GraphPad Prism 9.0 and GPower 3.0.10 software programs were used.

## 3 Results

**FIGURE 2:**
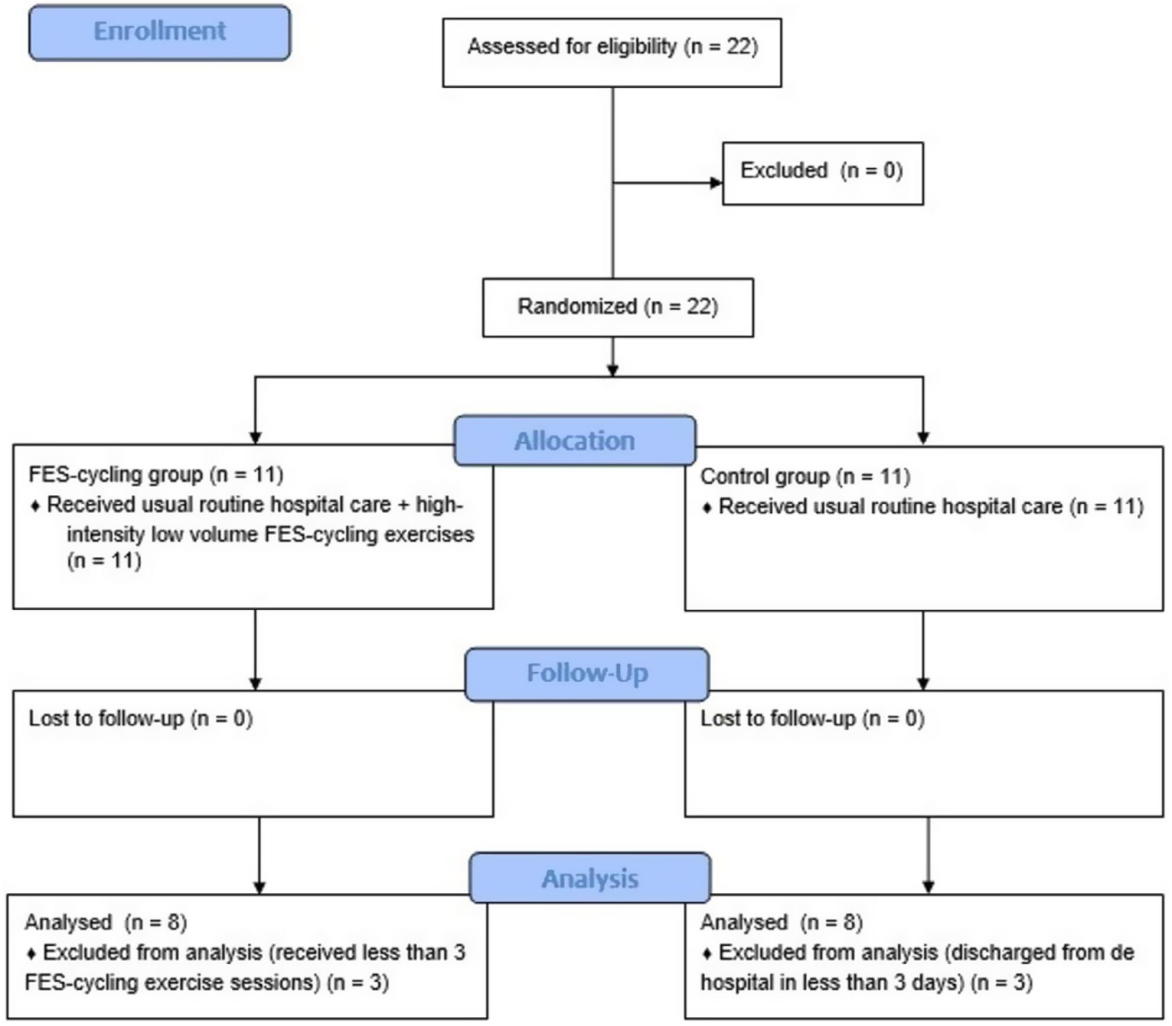
CONSORT flow diagram of participant enrollment, allocation, follow‐up, and analysis. [Color figure can be viewed at wileyonlinelibrary.com]

A total of 22 patients were enrolled in the study. None of them presented hemodynamic instability, resting tachycardia, or oxygen saturation < 90% even under oxygen therapy, or orthopedic limitations that prevented the FES‐cycling exercise. Six received < 3 FES‐cycling exercise sessions or were discharged from the hospital < 3 days after initial evaluation and were excluded from analysis (Figure 2). The characteristics of the remaining 16 patients (8 in each group) are described in Table 1. Intention to treat analysis was performed for muscle performance variables in one patient in each group.

**TABLE 1:** Basic characteristics, functional electrical stimulation parameters, muscle performance, ambulation capacity and primary organs disease of participants in FES‐cycling and control groups.

Variable	FES‐cycling (*n* = 8)	Control (*n* = 8)	*p*
Sex (male/female)	3/5	3/5	1.0
Age (years)	57 ± 18	54 ± 16	0.7626
Elapsed time between evaluations (days)	8 ± 4	8 ± 4	1.0
FES‐cycling sessions (*n*)	5 ± 2	—	—
**FES parameters used in evaluations**			
Current amplitude (mA)	94 ± 29	96 ± 47	0.8991
Pulse width (μs)	500 (500–600)	550 (500–600)	0.5431
**Muscle performance**			
Torque (Nm)	3.67 (2.75–4.55)	3.59 (2.90–5.51)	0.5737
Power (watts)	6.41 (5.62–7.78)	6.61 (5.82–7.48)	0.7983
Stimulation cost (×10^3^, μC/watt)	5.73 (4.66–12.17)	8.45 (2.70–13.82)	0.8785
**Ambulation capacity (*n*, %)**			
Bed restriction	6 (75)	6 (75)	1.0
Ambulation with support	2 (25)	2 (25)	1.0
Independent ambulation	0 (0)	0 (0)	1.0
**Primary organs diseases (*n*, %)**			
Liver	2 (25)	2 (25)	—
Hematological	1 (12.5)	0 (0)	—
Neurological	0 (0)	1 (12.5)	—
Pulmonary	3 (37.5)	1 (12.5)	—
Heart	1 (12.5)	2 (25)	—
Autoimmune	1 (12.5)	2 (25)	—

*Note:* Data are presented as mean ± standard deviation or median (interquartile range) for continuous variables and as frequency for categorical variables.

Abbreviations: FES, functional electrical stimulation; mA, milliamps; mm, millimeters; Nm, newton meters; μC, microcoulombs.

**FIGURE 3:**
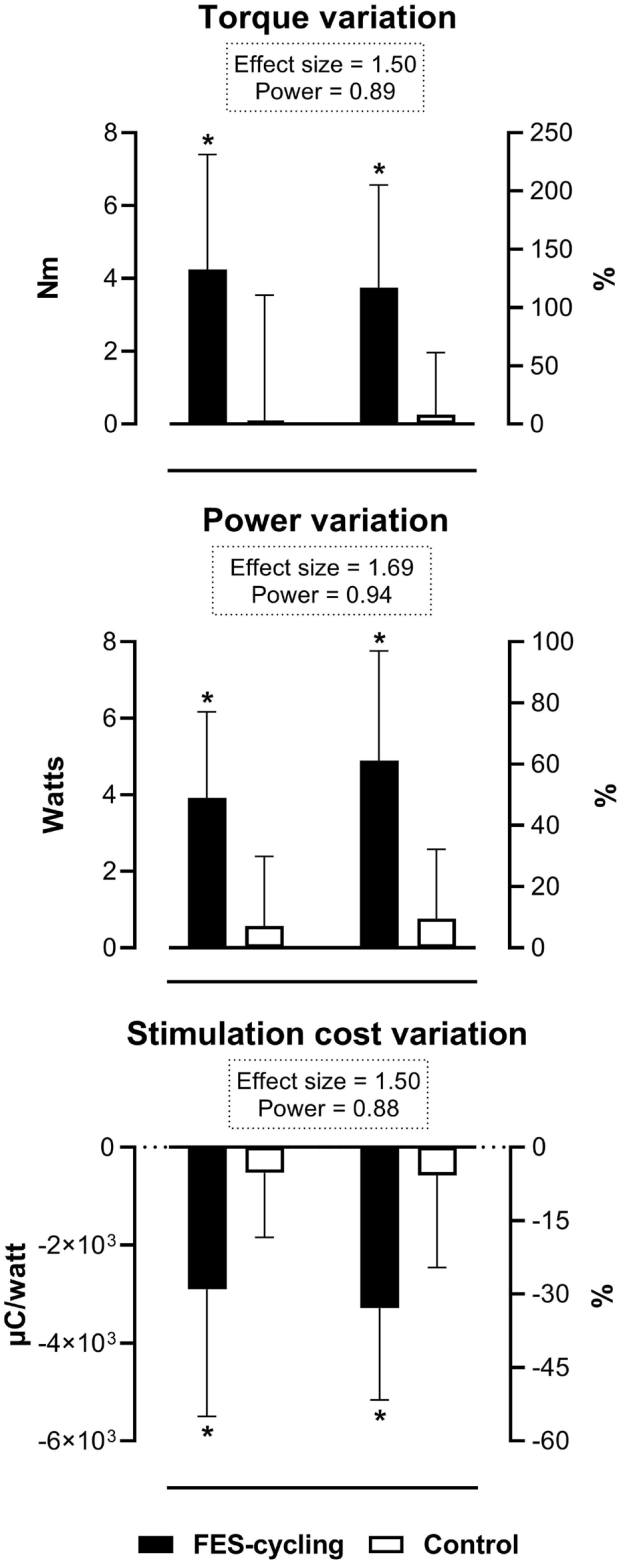
Variation in muscle performance after treatment. Data are presented as means ± standard deviations. *p < 0.05 indicates statistical significance.

The FES‐cycling group presented higher absolute (4.25 ± 3.15 vs. 0.04 ± 3.49 Nm, *p* = 0.024) and percentage (117 ± 88 vs. 8% ± 53%, *p* = 0.0096) torque variation compared to control, with a large effect size (effect size = 1.50, power = 0.89) (Figure 3). The FES‐cycling group presented higher absolute (3.91 ± 2.25 vs. 0.57 ± 1.82 watts, *p* = 0.0057) and percentage (61 ± 36 vs. 10% ± 23%, *p* = 0.0040) power variation compared to control, with a large effect size (effect size = 1.69, power = 0.94) (Figure 3). The FES‐cycling group also presented higher absolute (−2903 ± 2598 vs. −523 ± 1319 μC/watt, *p* = 0.0367) and percentage (−33 ± 18 vs. −6% ± 18%, *p* = 0.0119) stimulation cost variation compared to control, both with a large effect size (effect size = 1.50, power = 0.88) (Figure 3).

**FIGURE 4:**
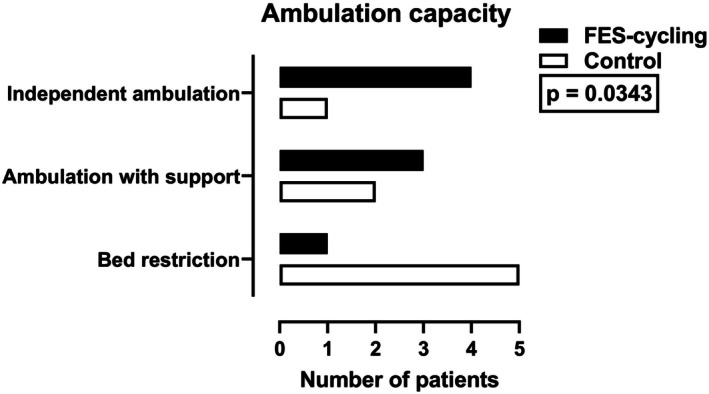
Ambulation capacity outcomes following treatment (the initial states of the participants are not presented).

Six patients improved their ambulation capacity in the FES‐cycling group compared to two in the control group (*p* = 0.0343). One patient in the FES‐cycling group was restricted to bed compared to five in the control group. Three patients in the FES‐cycling group presented ambulation with support compared to two in the control group, and independent ambulation was observed in four patients in the FES‐cycling group compared to one in the control group (Figure 4).

**FIGURE 5:**
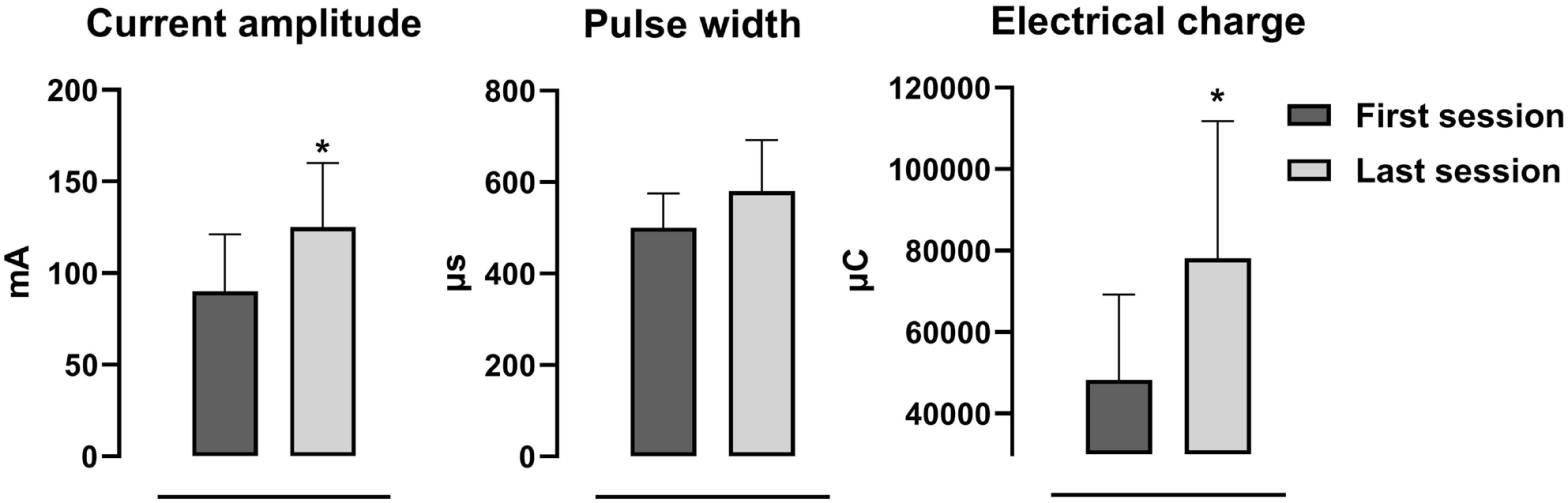
FES‐cycling exercise periodization. Data are presented as means ± standard deviations for pulse intensity and electrical charge and as median (interquartile range) for pulse width. *p < 0.05 indicates statistical significance.

The current amplitude (90 ± 31 vs. 125 ± 35 mA, *p* = 0.0020) and electrical charge (48 250 ± 20 981 vs. 78 138 ± 33 625 μC, *p* = 0.0121) increased from the first to the last session. The pulse width had a nonsignificant variation (500 [500–575] vs. 580 [500–692] μs, *p* = 0.1250) (Figure 5). At the first session, the current amplitude ranged from 60 to 150 mA and the pulse width from 500 to 600 μs. At the last session, the current amplitude ranged from 80 to 190 mA and the pulse width from 500 to 800 μs.

## 4 Discussion

Our study highlights significant findings: (1) Recumbent high‐intensity, low‐volume FES‐cycling exercise increased strength, power, and neuromuscular efficiency. (2) Ambulation capacity improved threefold in the FES‐cycling exercise group compared to the control, and (3) exercise periodization was primarily driven by increases in current amplitude.

At baseline, patients' muscle performance was below that of healthy subjects, and the torque values reached the critical illness weakness threshold [19]. Our recent systematic review with meta‐analysis supports these findings, showing enhanced torque and power with FES‐cycling [24]. A plausible physiological explanation is that high‐intensity FES‐cycling likely promotes substantial intramuscular tissue pressure and muscle tension induced by the electrical stimulation, inducing ischemia [25], which enhances metabolic stress [26] and triggers muscle adaptations.

Neuromuscular electrophysiological disorders alter the neuromuscular excitability threshold, and critically ill patients often present chronaxie ≥ 1000 μs [27]. Additionally, high current amplitudes are required in lower musculoskeletal quality patients [28]. Subcutaneous adipose tissue and intramuscular fat act as insulation against the propagation of current during surface electrical stimulation [29].

The observed enhancement in neuromuscular efficiency indicated by reduced stimulation cost, suggests that lower motor unit recruitment was required for the same power output, or higher power output was achieved for the same motor unit recruitment. Electrical stimulation has been shown to improve the force development rate effectively via neural factor activity [30]. The additional muscle tension induced by isokinetic cycling could potentially amplify these effects. Moreover, it is well‐established that neural adaptations precede structural adaptations. Thus, the observed increase in muscle force‐generating capacity is mainly due to neuromuscular adaptation rather than hypertrophy changes [31]. Visually, there were no noticeable changes in muscle trophism among the patients of the present study, further suggesting that the improvement in neuromuscular efficiency may have been due to neural adaptations.

Ambulation capacity was also improved, a natural consequence of enhanced muscle performance. The close relationship between torque variation and gait speed [32] is well documented, and early ambulation itself is known to promote strength gains [33], creating a perfect symbiosis between these outcomes. Research by Mateo et al. [34] demonstrated that patients engaging in FES‐cycling exercise increase their daily walking durations. Importantly, ambulation capacity is closely related to hospital length of stay and survival rates [18]. Importantly, ambulation capacity is closely linked to hospital length of stay and survival rates, underscoring its significance as a therapeutic target. Given these considerations, high‐intensity, low‐volume FES‐cycling may be the optimal modality for hospitalized patients who typically need to cover only short distances, such as moving from the bed to the bathroom or navigating around ward/ICU halls. Conversely, low‐intensity, high‐volume exercise regimes might be better suited for long‐term programs aimed at enhancing cardiorespiratory fitness.

To date, three randomized controlled trials have analyzed the effects of FES‐cycling exercise on hospitalized patients. However, none of them used a high‐intensity, low‐volume protocol. Waldauf et al. [12] used a very low‐intensity, high‐volume regimen, with current amplitude ranging from 0 to 60 mA, a frequency of 40 Hz, and a pulse width of 250 μs, with a mean of 31 min per day of exercise protocol. The patients received a mean of 6.5 FES‐cycling treatment days (similar to the present study), showing no muscle strength or physical function benefits compared to the control group. Berney et al. [11] used a low‐intensity, high‐volume FES‐cycling exercise protocol, with a current amplitude between 20 and 30 mA, a frequency range from 43.5 to 50 Hz, and pulse widths of 250 or 300 μs, with a median of 51 min per day and five sessions (similar to the present study). This group observed no benefits on muscle strength but reported a physical function 25% higher than in the control group. Parry et al. [6] used a moderate‐intensity, high‐volume FES‐cycling exercise protocol, with current amplitude up to 140 mA, frequency ranging from 30 to 50 Hz, and pulse width ranging from 300 to 400 μs, with a 20–60 min per day protocol. The patients received a mean of 8.6 FES‐cycling sessions (higher than in the present study), and the physical function was 83% better than in the control group.

In contrast to these studies, our research increased exercise intensity and reduced volume, aligning with emerging evidence suggesting a dose‐dependent relationship in the efficacy of FES‐cycling. The substantial improvements in muscle performance observed in our study, characterized by a large effect size, underscore the clinical relevance of adopting a high‐intensity, low‐volume regimen as the preferred approach for FES‐cycling exercise in hospitalized patients.

Once the safety of high‐intensity FES‐cycling parameters had been established in hospitalized patients [13], we strongly encouraged the patients to allow the physical therapist to gradually increase the electrical charge (current amplitude and/or pulse width) during each session. The patients were very well instructed that the session time would be short so that they could withstand such intensity. Some patients reported discomfort in the first/second minute of exercise but got used to the high intensity afterward. In general, the patients tolerated the 10‐min exercise session very well. No adverse events were recorded in 42 sessions.

Feelings of displeasure during exercise and delayed‐onset muscle soreness are two barriers that could impair the execution of high‐intensity exercise. However, it was not a significant obstacle during the present study. Dierkes et al. [35] demonstrated no association of training type (high or moderate intensities) and affective to exercise, suggesting that high‐intensity activities are as tolerable as moderate ones. Additionally, Farias Junior et al. [36] showed no difference in the magnitude of delayed‐onset muscle soreness after a high‐intensity, low‐volume or moderate‐intensity, high‐volume exercise. In the present study, the exercise intensity was well tolerated, and no patient reported a significant delayed‐onset muscle soreness that could impair the FES‐cycling exercise sessions.

### 4.1 Study Limitations

The present study has limitations that could impact the interpretation of the results: (1) limited evaluation methods. Ultrasound and electromyography evaluations were scheduled to determine muscle thickness and motor units' recruitment. However, these evaluations were not performed. The ultrasound equipment presented technical problems, and the electromyography evaluator was no longer available for the research. (2) The absence of monitoring at the hospital routine of physical therapy procedures. These procedures were performed by the hospital's physical therapy team. To avoid any interference as much as possible, the hospital team and researchers were blind to each other. (3) The reduced number of researchers available for FES‐cycling exercise sessions made it impossible to carry out a double‐masked study. To compensate for the single blind, very objective evaluation criteria were adopted to avoid any interference from the evaluator in the results. (4) High frequency. While it is well established that higher frequencies may contribute to premature fatigue, our choice of 75 Hz was carefully made to optimize motor unit recruitment during FES. In the context of a short‐duration exercise protocol (10 min), the risk of significant muscle fatigue is likely less of a concern compared to longer‐duration protocols (20–60 min), where fatigue would be more pronounced.

## 5 Conclusions

This study's findings demonstrate that recumbent high‐intensity, low‐volume FES‐cycling exercise significantly enhances strength, power, and neuromuscular efficiency in hospitalized patients and markedly improves their ambulation capacity. Given these outcomes, recumbent high‐intensity, low‐volume FES‐cycling emerges as a particularly effective exercise modality for hospitalized patients, suggesting its suitability over other training intensities for this specific patient population. This exercise regimen aligns with the physiological needs of hospitalized patients and efficiently addresses the practical constraints of hospital environments, maximizing therapeutic benefits within limited session durations.

## Author Contributions

Concept/design (M.F., F.L.M.), data analysis/interpretation (M.F., G.C.), drafting article (M.F.), critical revision of article (F.L.M., G.C.), approval of article (M.F., F.L.M., G.C.), statistics (M.F.), data collection (M.F., F.L.M.).

## Conflicts of Interest

Murillo Frazão serves as a technical consultant for a manufacturer of FES‐cycling equipment.
